# Serum Chloride Level Is Associated With Abdominal Aortic Calcification

**DOI:** 10.3389/fcvm.2021.800458

**Published:** 2022-01-18

**Authors:** Sheng Hu, Tian Lan, Silin Wang, Lang Su, Sheng Zou, Jiayue Ye, Yang Zhang, Deyuan Zhang, Qiang Guo, Wenxiong Zhang, Dongliang Yu, Jianjun Xu, Yiping Wei, Jinhua Peng

**Affiliations:** ^1^Department of Thoracic Surgery, The Second Affiliated Hospital of Nanchang University, Nanchang, China; ^2^Department of Health Care Management, The Second Affiliated Hospital of Nanchang University, Nanchang, China

**Keywords:** serum chloride, abdominal aortic calcification, association, multiple equation regression analysis, smooth curve fitting

## Abstract

**Background:**

Abdominal aortic calcification is a potentially important independent risk factor for cardiovascular health. The aim of this study was to determine the relationship between serum chloride level and abdominal artery calcification.

**Methods:**

We obtained the data of 3,018 individuals from the National Health and Nutrition Examination Survey database and analyzed the relationship between serum chloride and abdominal artery calcification. We performed stratified and single factor analysis, multiple equation regression analysis, smooth curve fitting, and threshold effect and saturation effect analysis. R and EmpowerStats were used for data analysis.

**Results:**

Serum chloride is independently related to the AAC total 24 score (AAC-24). The smooth curves fitted were all inverted-U shaped. Below a cutoff value of 92 mmol/L, increase in serum chloride level was associated with increase in AAC-24; however, above that cutoff, increase in serum chloride level was associated with decrease in AAC-24.

**Conclusions:**

At serum levels below 92 mmol/L, chloride is a risk factor for abdominal aortic calcification but levels above 92 mmol/L appear to protect against abdominal aortic calcification.

## Introduction

Aortic calcification is a risk marker for cardiovascular diseases such as coronary artery disease and stroke (1–4). Factors known to be strongly associated with abdominal aortic calcification (AAC) include old age, chronic kidney disease, sex, diabetes mellitus (5), and osteoporosis (6, 7). Several other factors are also probably involved; their identification is essential for effective prevention of AAC.

Serum chloride level has complex effects in humans. It is associated with several diseases, including hypertension (8), heart failure (9), acute pancreatitis (10), acute kidney injury (10), chronic kidney disease (11), pulmonary arterial hypertension (11), cardiorenal syndrome (12), and neuromyelitis optica (13). Changes in serum chloride level—especially increase in serum level—during hospitalization has been shown to be associated with increased in-hospital mortality in patients with chronic kidney disease and pulmonary arterial hypertension (11, 14). In addition, there are studies showing that lanthanum chloride influences bovine vascular smooth muscle cell calcification bi-directionally (15) and that magnesium chloride can block tissue valve calcification (16). However, the relationship between serum chloride and AAC in humans has not been well-studied. We speculate that serum chloride could be one such factor.

The aim of this study was to evaluate the relationship between serum chloride and abdominal aortic calcification using a representative sample of elderly individuals from the National Health and Nutrition Examination Survey (NHANES) (17).

## Materials and Methods

### Study Population

The study population was selected from the database of the NHANES—a population-based cross-sectional survey designed to collect information about the health and nutrition of the population of the US. The project surveys a nationally representative sample each year. These populations are located in counties across the country. NHANES interviews collect demographic, socioeconomic, dietary, and health-related information. Physical examinations conducted during the survey include physiological measurements and laboratory examinations (18).

**Figure 1:**
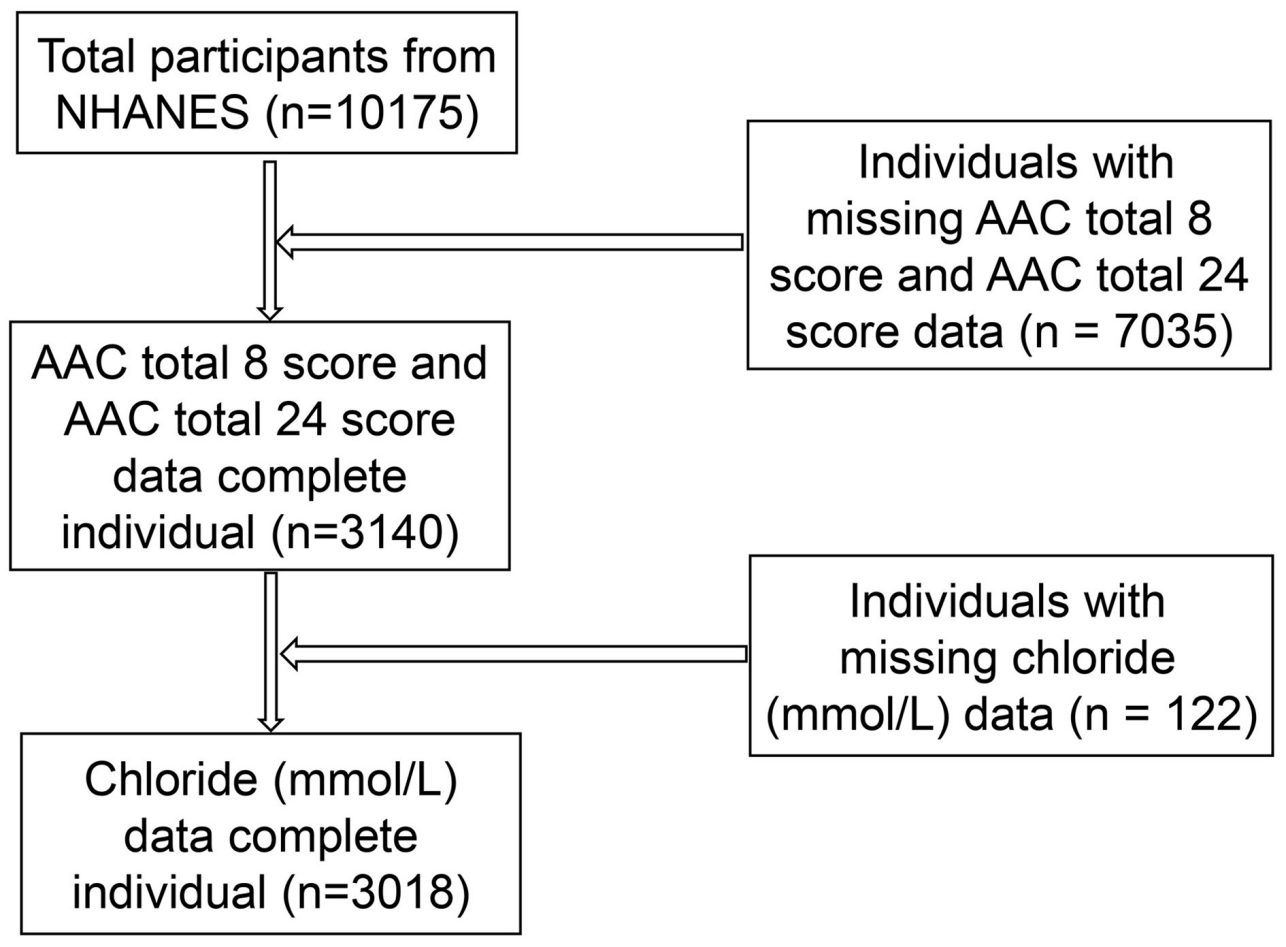
Flowchart of screening participants.

In this study, we retrospectively analyzed the data recorded for the period 2013–2014, which represents one cycle of the NHANES. From among the 10,175 individuals registered in the NHANES database during this period, we eliminated 7,035 because of missing data on AAC Total 24 score (AAC-24) and another 122 because of missing data on serum chloride level. The data of the remaining 3,018 individuals were included in this analysis. Figure 1 is a flowchart for screening participants.

### Ethics Statement

This study was approved by the Ethics Review Board of the National Center for Health Statistics. Informed consent was not considered necessary as this was an analysis of a database.

### Variables

The exposure variable for this study was serum chloride level (mmol/L). Blood specimens were processed and stored in the mobile examination center laboratory, and shipped to the Johns Hopkins University Lipoprotein Analytical Laboratory for analysis. The outcome variable was AAC-24, which was used to describe the amount of calcification. Several studies have shown that lateral spine images obtained with dual-energy X-ray absorptiometry (DXA) for vertebral fracture assessment can detect AAC with reasonably good sensitivity and specificity. The image resolution of lateral spine scans obtained with DXA is close that of a standard radiograph, and radiation exposure is much lower (19, 20). For scoring AAC-24, the anterior and posterior aortic walls were each divided into four segments, corresponding to the areas in front of lumbar vertebrae L1–L4. Within each of the eight segments, aortic calcification was recognized visually as either a diffused white stippling of the anterior and/or posterior aortic walls, or as white linear calcification of the anterior and/or posterior walls. Aortic calcification was scored as “0” if there was no calcification in that segment; “1” if one-third or less of the aortic wall in that segment was calcified; “2” if more than one-third but <two-thirds was calcified; or “3” if more than two-thirds was calcified. The scores were obtained separately for the anterior and posterior aortic wall; thus, the score at each vertebral level ranged from “0” to “6,” and the total score from “0” to “24” (20, 21), see Supplementary Table S1 for details of AAC-24 calculations.

The categorical variables included as covariates in our analysis were sex, race/ethnicity, and education level. The continuous covariates included in our analysis were age, body mass index (BMI), systolic blood pressure, diastolic blood pressure, total serum calcium (mmol/L), serum cholesterol (mmol/L), serum albumin (g/L), serum glucose, and refrigerated serum glucose (mmol/L). All data on serum chloride, AAC-24 score, and the covariates are available at http://www.cdc.gov/nchs/nhanes/.

### Statistical Analysis

We performed weighted and variance estimation analyses to account for significant variance in our data set. Weighted multiple logistic regression models were used to assess the association between serum chloride and AAC-24. Weighted chi-square test was used to analyze differences in categorical variables between groups, and a weighted linear regression model was used to analyze differences in continuous variables. Stratified multiple regression was used for subgroup analysis. In addition, smooth curve fitting and generalized additive models were used to address the non-linear relationship between serum chloride and AAC-24. A recursive algorithm was used to calculate the inflection point in the relationship between serum chloride and AAC-24 when non-linearity was detected, with a bi-segmented linear regression model on either side of the inflection point. Data analysis was performed using R 3.6.3 (http://www.r-project.org) and EmpowerStats (www.empowerstats.net, X&Y Solutions Inc., Boston, MA, USA). *P* < 0.05 was considered statistically significant.

## Results

Serum chloride was divided into three tertile groups: low: ≥87 mmol/L to 103 mmol/L; medium: ≥103 mmol/L to <105 mmol/L; and high: ≥105 mmol/L to <119 mmol/L. As Table 1 shows, there were significant differences in baseline characteristics between the three tertile groups. Mean age, systolic blood pressure, serum cholesterol, serum albumin, serum calcium, and serum glucose were all significantly higher in the low tertile group than in the other tertile groups. AAC-24 was lowest in the medium tertile group. Mean BMI was highest in the high tertile group. Race/ethnicity, sex, and diastolic blood pressure were not significantly different between the three tertile groups.

**Table 1:** Baseline characteristics of participants (*N* = 3,018).

**Chloride (mmol/L)**	**Total**	**Low**	**Middle**	**High**	***P*-value**
		**(≥87 to 103)**	**(≥103 to <105)**	**(≥105 to ≤119)**	
AAC total 24 score	1.473 ± 3.285	2.130 ± 3.931	1.112 ± 2.801	1.339 ± 3.120	<0.00001
Chloride (mmol/L)	104.044 ± 2.814	100.478 ± 2.052	103.528 ± 0.499	106.387 ± 1.475	<0.00001
Age (years)	57.425 ± 11.550	58.313 ± 11.730	56.888 ± 11.204	57.273 ± 11.640	0.03528
BMI (kg/m^2^)	28.541 ± 5.504	28.177 ± 5.285	28.116 ± 5.451	29.024 ± 5.619	0.00007
Systolic blood pressure (mmHg)	125.193 ± 17.671	127.551 ± 18.216	123.346 ± 17.565	125.024 ± 17.260	0.00002
Diastolic blood pressure (mmHg)	71.104 ± 12.334	71.835 ± 12.602	70.940 ± 11.216	70.788 ± 12.839	0.17506
Total calcium (mmol/L)	2.364 ± 0.090	2.385 ± 0.093	2.366 ± 0.077	2.349 ± 0.093	<0.00001
Cholesterol (mmol/L)	5.085 ± 1.107	5.203 ± 1.173	5.142 ± 1.109	4.982 ± 1.058	0.00001
Albumin (g/L)	42.508 ± 3.015	43.138 ± 3.350	42.809 ± 2.715	41.958 ± 2.901	<0.00001
Glucose, refrigerated serum (mmol/L)	5.913 ± 2.258	6.603 ± 3.570	5.651 ± 1.573	5.695 ± 1.464	<0.00001
Gender (%)					0.09330
Male	48.292	51.606	47.769	46.766	
Female	51.708	48.394	52.231	53.234	
Race/Hispanic origin (%)					0.58950
Mexican American	6.985	6.570	6.733	7.383	
Other Hispanic	4.596	4.361	4.233	4.965	
Non-Hispanic white	71.452	72.773	73.102	69.636	
Non-Hispanic black	9.775	9.399	8.353	10.911	
Other races (a)	7.192	6.897	7.578	7.106	
Education level (%)					0.04164
<9th grade	4.997	4.869	4.968	5.088	
9–11th grade (b)	10.229	9.114	9.600	11.265	
High school graduate (c)	21.784	22.156	20.383	22.486	
Some college or AA degree	30.041	30.944	27.488	31.194	
College graduate or above	32.95	32.917	37.562	29.969	

*(a) Including Multi-Racial; (b) Includes 12th grade with no diploma; (c) GED or equivalent. BMI, Body mass index. Weighted by: Full sample mobile examination center exam weight. Mean +/- standard deviation for: AAC total 24 score, chloride, age, BMI, systolic blood pressure, diastolic blood pressure, total calcium, cholesterol, albumin, glucose, refrigerated serum. P value was calculated using weighted linear regression model. % For: gender, race/ethnicity, race/Hispanic origin, education level. P value was calculated using weighted chi-square test*.

Table 2 shows the results of univariate analysis. Beta value >0 indicates that the factor is a risk factor for AAC, and beta value <0 indicates that the factor is a protective factor. Compared with the low tertile group of serum chlorine levels, the β value (CI) of the serum chlorine level middle tertile group is −1.017 (−1.333, −0.702), and the β value of the serum chlorine level high tertile group is −0.791 (−1.079, −0.502). *P* values are <0.001. The beta value, *P* value, 95% confidence interval and P for trend value of different covariates are shown in Table 2 (gender, age, race/Hispanic origin, education level recoded, BMI, systolic blood pressure, diastolic blood pressure, total calcium, cholesterol, albumin, glucose, refrigerated serum).

**Table 2:** Univariate analysis for AAC total 24 score.

	**Statistics (%)**	**AAC total 24 score (β (95%CI) *P*-value)**
Chloride (mmol/L) tertiles
≧87 to 103	773 (25.613%)	0
≧103 to <105	851 (28.197%)	−1.017 (−1.333, −0.702) <0.00001
≧105 to ≦119	1,394 (46.190%)	−0.791 (−1.079, −0.502) <0.00001
P for trend		<0.001
Gender
Male	1,454 (48.178%)	0
Female	1,564 (51.822%)	0.138 (−0.097, 0.372) 0.25020
P for trend		0.250
Age (years) tertiles
40–50	942 (31.213%)	0
51–63	1,009 (33.433%)	0.574 (0.314, 0.835) 0.00002
64–80	1,067 (35.355%)	2.905 (2.632, 3.179) <0.00001
P for trend		<0.001
Race/Hispanic origin
Mexican American	400 (13.254%)	0
Other Hispanic	286 (9.476%)	0.158 (−0.544, 0.861) 0.65815
Non-Hispanic white	1,339 (44.367%)	0.785 (0.322, 1.249) 0.00090
Non-Hispanic black	577 (19.119%)	0.191 (−0.388, 0.770) 0.51888
Other races (a)	416 (13.784%)	0.498 (−0.123, 1.119) 0.11602
P for trend		0.339
Education level recoded		
<9th grade	282 (9.344%)	0
9–11th grade (b)	407 (13.486%)	0.031 (−0.607, 0.668) 0.92446
High school graduate (c)	683 (22.631%)	0.080 (−0.499, 0.660) 0.78611
Some college or AA degree	851 (28.197%)	−0.206 (−0.770, 0.358) 0.47470
College graduate or above	795 (26.342%)	−0.657 (−1.218, −0.096) 0.02174
P for trend		<0.001
BMI (kg/m^2^) tertiles		
14.200–25.700	990 (33.044%)	0
25.800–30.200	1,007 (33.611%)	0.072 (−0.217, 0.360) 0.62669
30.300–51.200	999 (33.344%)	−0.469 (−0.757, −0.181) 0.00143
P for trend		0.001
Systolic blood pressure (mmHg) tertiles
74–116	899 (32.490%)	0
118–130	860 (31.081%)	0.272 (−0.018, 0.563) 0.06639
132–228	1,008 (36.429%)	1.484 (1.193, 1.775) <0. 00001
P for trend		<0.001
Diastolic blood pressure (mmHg) tertiles
0–64	760 (27.467%)	0
66–74	981 (35.454%)	−0.929 (−1.234, −0.623) <0.00001
76–122	1,026 (37.080%)	−1.395 (−1.699, −1.091) <0.00001
P for trend		<0.001
Total calcium (mmol/L) tertiles
1.900–2.300	859 (28.643%)	0
2.325–2.375	1,009 (33.645%)	0.148 (-0.152, 0.448) 0.33342
2.400–3.000	1,131 (37.713%)	0.445 (0.151, 0.739) 0.00302
P for trend		0.002
Cholesterol (mmol/L) tertiles
2.069–4.551	1,000 (33.156%)	0
4.577–5.456	997 (33.057%)	−0.571 (−0.860, −0.283) 0.00011
5.482–16.525	1,019 (33.786%)	−0.516 (−0.804, −0.229) 0.00043
P for trend		<0.001
Albumin (g/L) tertiles
24.000–40.000	800 (26.508%)	0
41.000–43.000	1,187 (39.331%)	−0.033 (−0.339, 0.274) 0.83492
44.000–54.000	1,031 (34.162%)	−0.200 (−0.509, 0.110) 0.20581
P for trend		0.174
Glucose, refrigerated serum (mmol/L) tertiles
2.720–5.050	955 (31.643%)	0
5.110–5.770	1,048 (34.725%)	0.119 (−0.161, 0.399) 0.40529
5.830–32.030	1,015 (33.632%)	0.946 (0.655, 1.237) <0.00001
P for trend		<0.001

*(a) Including Multi-Racial; (b) Includes 12th grade with no diploma; (c) GED or equivalent. Weighted by: Full sample mobile examination center exam weight. AAC, Abdominal Aortic Calcification; CI, confidence interval; BMI, Body mass index*.

Table 3 shows the results of stratified analysis between serum chloride and AAC-24. The medium and high serum chloride groups were all negative by using the low serum chloride group as a reference. The absolute value of beta for female in the middle and high serum chloride groups were higher than those for male. The absolute value of beta was highest in the other Hispanic group, followed by the non-Hispanic White group and the Mexican-American group. In the stratification of different education levels, the absolute value of beta was highest in the <9th grade group. In the age stratification, the 64–80-year age-group had the highest absolute value of beta, which was significantly higher than the other age-groups. The absolute value of beta was maximum in the 0–64 mmHg diastolic blood pressure group. The absolute value of beta was maximum in the 1.9–2.3 mmol/L total calcium group. The absolute value of beta was maximum in the 4.577–5.456 mmol/L total cholesterol group. The absolute value of beta was maximum in the 24–40 g/L serum albumin group. The absolute value of beta was maximum in the 5.11–5.77 mmol/L refrigerated serum glucose group.

**Table 3:** Stratified analysis between Chloride (mmol/L) and AAC total 24 score.

**Y = AAC total 24 score**	**Serum chloride tertiles**		
**X = Chloride (mmol/L)**	**Low**	**Middle**	**High**
		**β (95%CI) *P* value**	**β (95%CI) *P* value**
Gender
Male	Reference	−0.990 (−1.412, −0.569) <0.0001	−0.691 (−1.077, −0.305) 0.0005
Female	Reference	−1.063 (−1.531, −0.595) <0.0001	−0.904 (−1.333, −0.476) <0.0001
Race/Hispanic origin
Mexican American	Reference	−0.681 (−1.272, −0.090) 0.0244	−0.656 (−1.189, −0.123) 0.0163
Other Hispanic	Reference	−1.482 (−2.274, −0.690) 0.0003	−1.633 (−2.337, −0.930) <0.0001
Non-Hispanic white	Reference	−1.129 (−1.628, −0.630) <0.0001	−0.850 (−1.311, −0.388) 0.0003
Non-Hispanic black	Reference	−0.133 (−0.760, 0.494) 0.6777	−0.274 (−0.818, 0.269) 0.3231
Other races (a)	Reference	−1.041 (−1.804, −0.279) 0.0077	−0.175 (−0.884, 0.534) 0.6289
Education level
<9th grade	Reference	−1.391 (−2.519, −0.263) 0.0163	−1.748 (−2.777, −0.718) 0.0010
9–11th grade (b)	Reference	−1.135 (−2.096, −0.174) 0.0211	−0.622 (−1.481, 0.236) 0.1562
High school graduate (c)	Reference	−0.976 (−1.717, −0.235) 0.0101	−1.079 (−1.742, −0.416) 0.0015
Some college or AA degree	Reference	−1.161 (−1.779, −0.544) 0.0002	−0.939 (−1.488, −0.390) 0.0008
College graduate or above	Reference	−0.747 (−1.251, −0.243) 0.0038	−0.414 (−0.897, 0.069) 0.0934
BMI (kg/m^2^) tertiles
14.200–25.700	Reference	−1.581 (−2.121, −1.041) <0.0001	−0.760 (−1.282, −0.239) 0.0044
25.800–30.200	Reference	−0.825 (−1.403, −0.247) 0.0053	−0.419 (−0.945, 0.106) 0.1180
30.300–51.200	Reference	−0.545 (−1.059, −0.031) 0.0380	−1.016 (−1.467, −0.566) <0.0001
Age (years) tertiles
40–50	Reference	−0.238 (−0.440, −0.036) 0.0211	−0.121 (−0.307, 0.066) 0.2042
51–63	Reference	−0.676 (−1.033, −0.320) 0.0002	−0.658 (−0.987, −0.328) <0.0001
64–80	Reference	−1.836 (−2.619, −1.052) <0.0001	−1.378 (−2.080, −0.677) 0.0001
Systolic blood pressure (mmHg) tertiles
74–116	Reference	−0.794 (−1.220, −0.367) 0.0003	−0.480 (−0.883, −0.076) 0.0200
118–130	Reference	−0.928 (−1.451, −0.404) 0.0005	−0.923 (−1.403, −0.442) 0.0002
132–228	Reference	−0.747 (−1.438, −0.057) 0.0342	−0.863 (−1.456, −0.270) 0.0045
Diastolic blood pressure (mmHg) tertiles
0–64.000	Reference	−2.141 (−2.958, −1.323) <0.0001	−1.925 (−2.662, −1.188) <0.0001
66.000–74.000	Reference	−0.943 (−1.481, −0.404) 0.0006	−0.540 (−1.036, −0.043) 0.0333
76.000–122.000	Reference	−0.339 (−0.698, 0.021) 0.0655	−0.508 (−0.836, −0.180) 0.0025
Total calcium (mmol/L) tertiles
1.900–2.300	Reference	−1.227 (−1.827, −0.627) <0.0001	−1.013 (−1.533, −0.492) 0.0001
2.325–2.375	Reference	−0.822 (−1.348, −0.296) 0.0023	−0.618 (−1.107, −0.128) 0.0136
2.400–3.000	Reference	−1.039 (−1.566, −0.512) 0.0001	−0.676 (−1.187, −0.165) 0.0097
Cholesterol (mmol/L) tertiles
2.069–4.551	Reference	−1.009 (−1.688, −0.330) 0.0037	−0.715 (−1.314, −0.117) 0.0193
4.577–5.456	Reference	−1.320 (−1.788, −0.851) <0.0001	−1.264 (−1.699, −0.829) <0.0001
5.482–16.525	Reference	−0.758 (−1.242, −0.273) 0.0022	−0.506 (−0.961, −0.051) 0.0296
Albumin (g/L) tertiles
24.000–40.000	Reference	−1.671 (−2.339, −1.003) <0.0001	−1.300 (−1.872, −0.728) <0.0001
41.000–43.000	Reference	−1.305 (−1.870, −0.740) <0.0001	−1.045 (−1.564, −0.526) <0.0001
44.000–54.000	Reference	−0.569 (−1.028, −0.111) 0.0151	−0.488 (−0.937, −0.040) 0.0332
Glucose, refrigerated serum (mmol/L) tertiles
2.720–5.050	Reference	−0.979 (−1.461, −0.497) <0.0001	−0.325 (−0.773, 0.123) 0.1557
5.110–5.770	Reference	−0.998 (−1.487, −0.510) <0.0001	−1.157 (−1.604, −0.711) <0.0001
5.830–32.030	Reference	−0.897 (−1.545, −0.250) 0.0067	−0.661 (−1.247, −0.075) 0.0274

*(a) Including Multi-Racial; (b) Includes 12th grade with no diploma; (c) GED or equivalent. BMI, Body mass index. Weighted by: Full sample mobile examination center exam weight*.

Table 4 shows the results of the multiple regression equation analysis. The outcome variable was AAC-24, and the exposure variable was serum chloride. Weighted by: Full sample mobile examination center examination. The crude model was unadjusted; model I adjusted for age, sex, and race/Hispanic origin; model II adjusted for age, sex, race/Hispanic origin, education level, BMI, systolic blood pressure, diastolic blood pressure, total calcium, cholesterol, albumin, and refrigerated serum glucose; and model III adjusted for age (smooth), sex, race/Hispanic origin, education level, BMI (smooth), systolic blood pressure (smooth), diastolic blood pressure (smooth), total calcium (smooth), cholesterol (smooth), albumin (smooth), and refrigerated serum glucose (smooth). The beta value for model III was −0.110. We stratified by chloride tertiles and, additionally, by the following covariates: sex, age, race/Hispanic origin, education level, BMI, systolic blood pressure, diastolic blood pressure, total calcium, cholesterol, albumin, and adjusted serum glucose (Table 4).

**Table 4:** Relationship between serum chloride and abdominal aortic calcification (multiple regression equation analysis).

**Outcome**	**Crude model**	**Model I**	**Model II**	**Model III**
	**β** **(95%CI) P-value**	**β** **(95%CI) P-value**	**β** **(95%CI) P-value**	**β** **(95%CI) P-value**
Total	−0.170 (−0.250, −0.091) 0.00003	−0.126 (−0.199, −0.053) 0.00074	−0.099 (−0.175, −0.023) 0.01116	−0.110 (-0.185,−0.036) 0.00383
Chloride (mmol/L) tertiles	
≧87 to 103	−0.219 (−0.353, −0.085) 0.00146	−0.092 (−0.213, 0.030) 0.13823	−0.076 (−0.206, 0.054) 0.25306	−0.096 (-0.225, 0.033) 0.14598
≧103 to <105	0.037 (−0.341, 0.414) 0.84873	−0.090 (−0.443, 0.262) 0.61545	−0.076 (−0.456, 0.305) 0.69740	0.006 (-0.352, 0.363) 0.97578
≧105 to ≦119	−0.133 (−0.244, −0.022) 0.01895	−0.130 (−0.232, −0.028) 0.01251	−0.117 (−0.221, −0.012) 0.02872	−0.115 (-0.217,−0.013) 0.02676
Gender	
Male	−0.108 (−0.167, −0.050) 0.00026	−0.131 (−0.184, −0.077) <0.00001	−0.110 (−0.169, −0.052) 0.00024	−0.130 (-0.188,−0.073) 0.00001
Female	−0.153 (−0.211, −0.094) <0.00001	−0.084 (−0.138, −0.030) 0.00235	−0.084 (−0.142, −0.026) 0.00434	−0.074 (-0.130,−0.017) 0.01062
Age (years) tertiles	
40–50	−0.028 (−0.056, 0.001) 0.05629	−0.029 (−0.058, −0.001) 0.04592	−0.029 (−0.061, 0.003) 0.07154	−0.036 (-0.069,−0.004) 0.02966
51–63	−0.116 (−0.164, −0.068) <0.00001	−0.117 (−0.165, −0.069) <0.00001	−0.107 (−0.160, −0.054) 0.00009	−0.113 (-0.166,−0.060) 0.00003
64–80	−0.189 (−0.283, −0.096) 0.00007	−0.185 (−0.279, −0.091) 0.00012	−0.162 (−0.259, −0.064) 0.00117	−0.173 (-0.270,−0.076) 0.00049
Race/Hispanic origin	
Mexican American	−0.100 (−0.175, −0.025) 0.00904	−0.088 (−0.162, −0.015) 0.01854	−0.117 (−0.200, −0.034) 0.00585	−0.114 (-0.196,−0.031) 0.00716
Other Hispanic	−0.236 (−0.336, −0.136) <0.00001	−0.226 (−0.323, −0.128) <0.00001	−0.213 (−0.325, −0.101) 0.00025	−0.203 (-0.309,−0.097) 0.00022
Non-Hispanic white	−0.143 (−0.209, −0.076) 0.00003	−0.122 (−0.182, −0.062) 0.00007	−0.106 (−0.169, −0.042) 0.00117	−0.108 (-0.170,−0.045) 0.00082
Non-Hispanic black	−0.062 (−0.137, 0.012) 0.10317	−0.041 (−0.112, 0.031) 0.26509	−0.087 (−0.167, −0.007) 0.03282	−0.070 (-0.150, 0.010) 0.08795
Other races (a)	−0.030 (−0.141, 0.081) 0.59744	0.001 (−0.101, 0.103) 0.98274	0.066 (−0.047, 0.179) 0.25466	0.068 (-0.040, 0.176) 0.21821
Education level				
<9th grade	−0.172 (−0.310, −0.035) 0.01426	−0.179 (−0.302, −0.057) 0.00434	−0.140 (−0.288, 0.008) 0.06434	−0.128 (-0.267, 0.012) 0.07474
9–11th grade (b)	−0.069 (−0.184, 0.046) 0.24018	0.007 (−0.099, 0.113) 0.89763	0.043 (−0.073, 0.158) 0.46801	0.003 (-0.111, 0.116) 0.96447
High school graduate (c)	−0.217 (−0.315, −0.119) 0.00002	−0.197 (−0.287, −0.108) 0.00002	−0.231 (−0.326, −0.136) <0.00001	−0.237 (-0.330,−0.143) <0.00001
Some college or AA degree	−0.155 (−0.230, −0.081) 0.00005	−0.121 (−0.191, −0.052) 0.00067	−0.097 (−0.172, −0.022) 0.01140	−0.101 (-0.175,−0.027) 0.00768
College graduate or above	−0.066 (−0.141, 0.008) 0.08267	−0.067 (−0.135, 0.000) 0.05176	−0.058 (−0.132, 0.015) 0.12143	−0.072 (-0.140,−0.004) 0.03872
BMI (kg/m^2^) tertiles	
14.200–25.700	−0.157 (−0.234, −0.081) 0.00006	−0.133 (−0.202, −0.064) 0.00016	−0.148 (−0.218, −0.078) 0.00004	−0.138 (-0.205,−0.070) 0.00007
25.800–30.200	−0.095 (−0.171, −0.020) 0.01370	−0.081 (−0.150, −0.011) 0.02268	−0.084 (−0.161, −0.007) 0.03195	−0.099 (-0.175,−0.022) 0.01212
30.300–51.200	−0.129 (−0.193, −0.066) 0.00007	−0.106 (−0.166, −0.046) 0.00052	−0.086 (−0.152, −0.020) 0.01088	−0.093 (-0.157,−0.029) 0.00444
Systolic blood pressure (mm Hg) tertiles	
74–116	−0.081 (−0.145, −0.018) 0.01223	−0.062 (−0.120, −0.003) 0.04020	−0.054 (−0.116, 0.009) 0.09191	−0.057 (-0.119, 0.005) 0.07207
118–130	−0.156 (−0.224, −0.088) <0.00001	−0.156 (−0.221, −0.092) <0.00001	−0.117 (−0.184, −0.050) 0.00064	−0.087 (-0.153,−0.022) 0.00892
132–228	−0.130 (−0.210, −0.050) 0.00147	−0.117 (−0.191, −0.043) 0.00191	−0.114 (−0.191, −0.037) 0.00402	−0.129 (-0.206,−0.051) 0.00112
Diastolic blood pressure (mm Hg) tertiles	
0–64.000	−0.315 (−0.419, −0.211) <0.00001	−0.250 (−0.344, −0.155) <0.00001	−0.231 (−0.331, −0.132) <0.00001	−0.243 (-0.340,−0.146) <0.00001
66.000–74.000	−0.075 (−0.147, −0.003) 0.04212	−0.062 (−0.129, 0.005) 0.06895	−0.047 (−0.115, 0.021) 0.17673	−0.040 (-0.108, 0.027) 0.24469
76.000–122.000	−0.076 (−0.124, −0.029) 0.00172	−0.071 (−0.116, −0.026) 0.00208	−0.052 (−0.102, −0.003) 0.03807	−0.053 (-0.102,−0.005) 0.03246
Total calcium (mmol/L) tertiles	
1.900–2.300	−0.123 (−0.197, −0.048) 0.00132	−0.106 (−0.174, −0.038) 0.00235	−0.069 (−0.141, 0.003) 0.05961	−0.082 (-0.152,−0.013) 0.02061
2.325–2.375	−0.100 (−0.169, −0.031) 0.00476	−0.091 (−0.155, −0.027) 0.00575	−0.099 (−0.167, −0.031) 0.00460	−0.088 (−0.156, −0.021) 0.01034
2.400–3.000	−0.154 (−0.228, −0.080) 0.00004	−0.121 (−0.188, −0.055) 0.00038	−0.111 (−0.184, −0.039) 0.00261	−0.137 (-0.208,−0.066) 0.00017
Cholesterol (mmol/L) tertiles	
2.069–4.551	−0.095 (−0.176, −0.014) 0.02161	−0.052 (−0.125, 0.021) 0.16539	−0.035 (−0.114, 0.043) 0.38012	−0.036 (-0.113, 0.041) 0.36427
4.577–5.456	−0.198 (−0.262, −0.135) <0.00001	−0.173 (−0.231, −0.114) <0.00001	−0.168 (−0.229, −0.107) <0.00001	−0.163 (-0.223,−0.103) <0.00001
5.482–16.525	−0.121 (−0.189, −0.053) 0.00053	−0.104 (−0.168, −0.040) 0.00145	−0.101 (−0.171, −0.031) 0.00485	−0.106 (-0.174,−0.037) 0.00261
Albumin (g/L) tertiles	
24.000–40.000	−0.143 (−0.221, −0.065) 0.00033	−0.112 (−0.184, −0.041) 0.00219	−0.126 (−0.205, −0.047) 0.00175	−0.119 (-0.197,−0.040) 0.00306
41.000–43.000	−0.140 (−0.212, −0.067) 0.00016	−0.101 (−0.167, −0.034) 0.00299	−0.058 (−0.128, 0.012) 0.10603	−0.056 (-0.125, 0.012) 0.10476
44.000–54.000	−0.143 (−0.210, −0.075) 0.00004	−0.112 (−0.174, −0.049) 0.00046	−0.118 (−0.185, −0.052) 0.00052	−0.128 (-0.194,−0.061) 0.00017
Glucose, refrigerated serum (mmol/L) tertiles			
2.720–5.050	−0.092 (−0.158, −0.026) 0.00627	−0.082 (−0.143, −0.021) 0.00814	−0.100 (−0.162, −0.039) 0.00150	−0.083 (-0.142,−0.025) 0.00549
5.110–5.770	−0.160 (−0.224, −0.097) <0.00001	−0.133 (−0.192, −0.074) 0.00001	−0.130 (−0.193, −0.067) 0.00006	−0.134 (-0.198,−0.070) 0.00005
5.830–32.030	−0.108 (−0.190, −0.025) 0.01038	−0.095 (−0.170, −0.020) 0.01305	−0.107 (−0.186, −0.028) 0.00819	−0.103 (-0.183,−0.023) 0.01171

*(a) Including Multi-Racial; (b) Includes 12th grade with no diploma; (c) GED or equivalent. Abbreviations: CI, confidence interval; ACC, abdominal aortic calcification; BMI, Body mass index. Weighted by: Full sample mobile examination center exam weight. Outcome variable: AAC total 24 score. Exposure variable: serum chloride. The crude model is not adjusted. Model I adjusted for age, gender, and race/Hispanic origin. Model II adjusted for age, gender, race/Hispanic origin, education level, BMI, systolic blood pressure, diastolic blood pressure, total calcium, cholesterol, albumin and refrigerated serum glucose. Model III adjusted for age (smooth), gender, race/Hispanic origin, education level, BMI (smooth), systolic blood pressure (smooth), diastolic blood pressure (smooth), total calcium (smooth), cholesterol (smooth), albumin (smooth) and refrigerated serum glucose (smooth)*.

**Figure 2:**
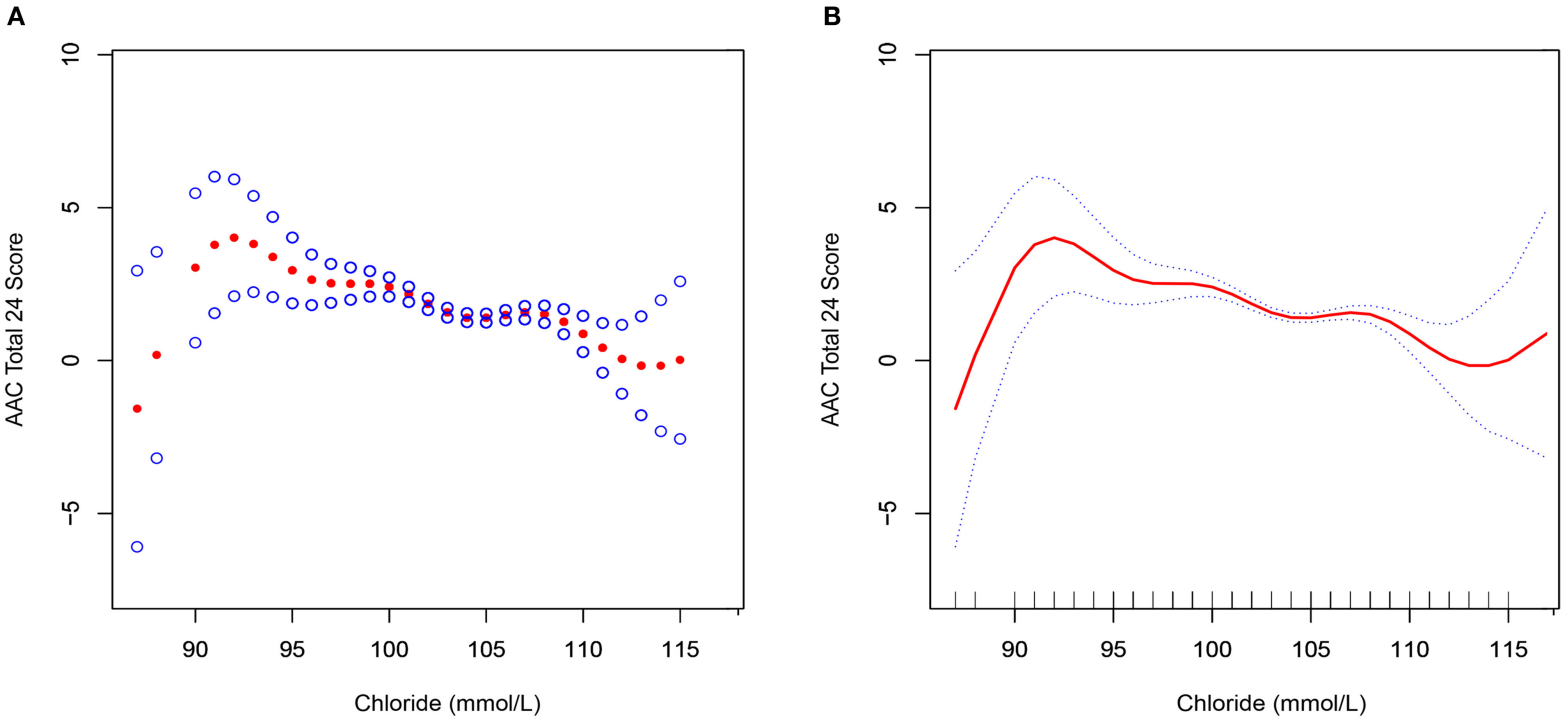
Association between serum chloride and abdominal aortic calcification. The red line represents the smooth curve fit between the variables. The blue bar represents the 95% confidence interval of the fit. Weighted by: full sample mobile examination center examination weight. Adjusted for age (smooth), sex, race/Hispanic origin, education level, BMI (smooth), systolic blood pressure (smooth), diastolic blood pressure (smooth), total calcium (smooth), cholesterol (smooth), albumin (smooth), and refrigerated serum glucose (smooth). **(A)** Scatter plot of curve fit. **(B)** Solid line plot of curve fit.

**Figure 3:**
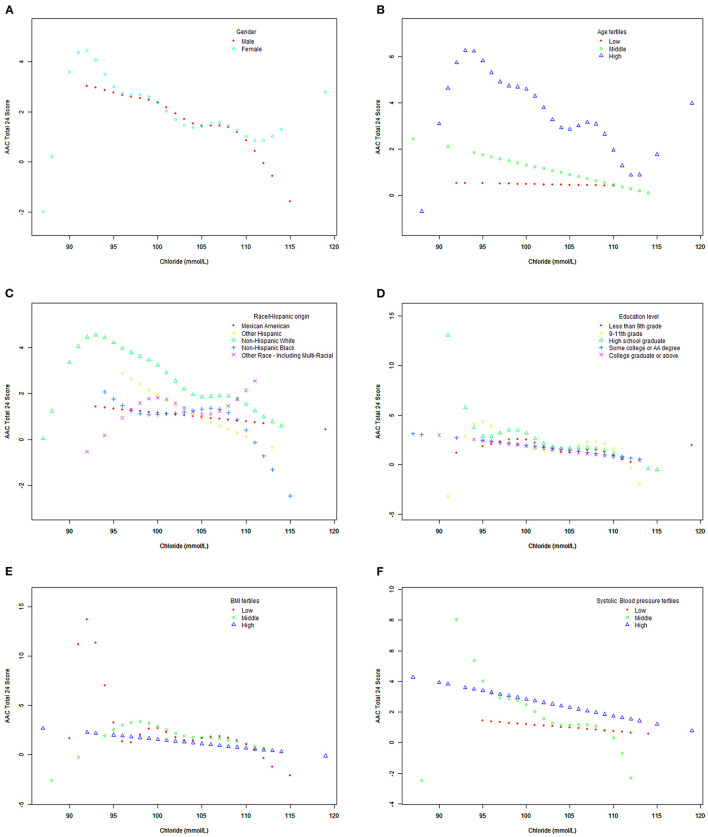
Association between serum chloride and abdominal aortic calcification, stratified by covariates (sex, age, race/Hispanic origin, education level, BMI, systolic blood pressure). Weighted by: full sample mobile examination center examination weight. Adjusted for age (smooth), sex, race/Hispanic origin, education level, BMI (smooth), systolic blood pressure (smooth), diastolic blood pressure (smooth), total calcium (smooth), cholesterol (smooth), albumin (smooth), and refrigerated serum glucose (smooth). **(A)** Stratified by sex. **(B)** Stratified by age tertiles. **(C)** Stratified race/Hispanic origin. **(D)** Stratified by education level. **(E)** Stratified by BMI tertiles. **(F)** Stratified by systolic blood pressure tertiles.

**Figure 4:**
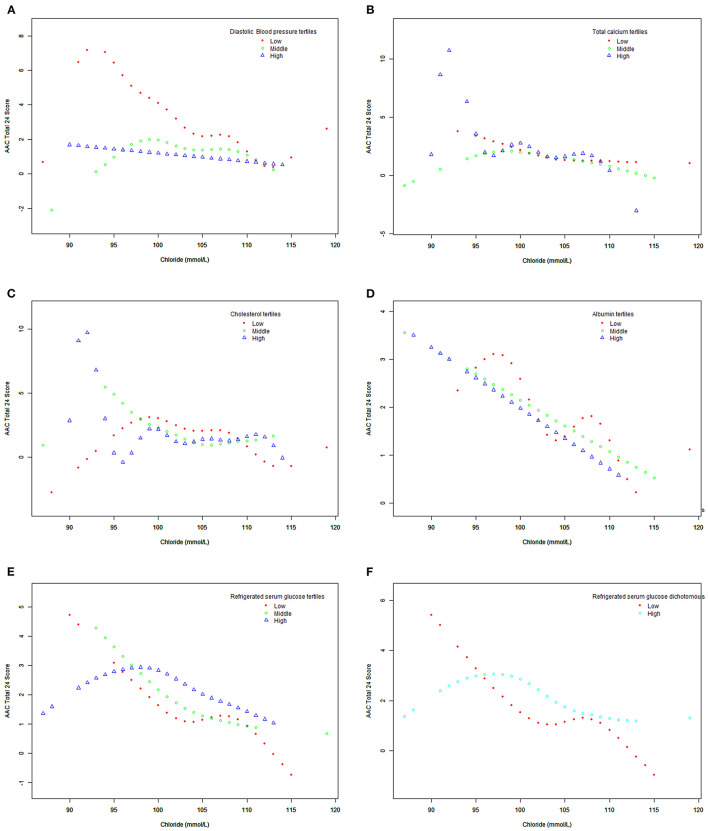
Association between serum chloride and abdominal aortic calcification, stratified by covariates (diastolic blood pressure, total calcium, cholesterol, albumin, refrigerated serum glucose). Weighted by: full sample mobile examination center exam weight. Adjusted for age (smooth), sex, race/Hispanic origin, education level, BMI (smooth), systolic blood pressure (smooth), diastolic blood pressure (smooth), total calcium (smooth), cholesterol (smooth), albumin (smooth), and refrigerated serum glucose (smooth). **(A)** Stratified by diastolic blood pressure tertiles. **(B)** Stratified by total calcium tertiles. **(C)** Stratified cholesterol tertiles. **(D)** Stratified by albumin tertiles. **(E)** Stratified by refrigerated serum glucose tertiles. **(F)** Stratified by refrigerated serum glucose (dichotomous).

Figure 2 shows the smooth fitting curves of serum chloride and AAC-24. The curve increases first and then decreases gradually, with inflection point being at serum chloride level of 92 mmol/L. We also analyzed the threshold effect and saturation effect using serum chloride of 92 mmol/L as the fold point (Table 5). Weighted by: Full sample mobile examination center exam weight. Outcome variable: AAC-24. Exposure variable: serum chloride (mmol/L). Table 5 shows the adjustment covariates 5. Model I showed a straight-line effect, with a beta value of −0.099 and *P* < 0.0001. Model II: serum chloride equal to 92 mmol/L was used as the fold point, and the results were statistically significant. Log likelihood ratio tests 0.003. The curve rose after 113 mmol/L. We performed the threshold effect and saturation effect analysis with 113, 114, and 115 mmol/L as folding points; the results are presented in Supplementary Tables S2–S4, respectively. However, the *P* values after folding point were all >0.05. Figures 3, 4 show the smoothed fitted curves of serum chloride and AAC-24, stratified by different covariates. The curves differed across different strata of individual covariates.

**Table 5:** Analysis of threshold effect and saturation effect with 92 mmol/L as cutoff serum chloride value point.

**Outcome**	**AAC total 24 score**
	**β (95%CI) *P*-value**
Model I	
A straight-line effect	−0.099 (−0.139, −0.059) <0.0001
Model II	
Fold points (K)	92
< K-segment effect 1	1.292 (0.430, 2.154) 0.0033
>K-segment effect 2	−0.114 (−0.155, −0.073) <0.0001
Effect size difference of 2 vs. 1	−1.406 (−2.276, −0.536) 0.0015
Equation predicted values at break points	3.257 (2.710, 3.803)
Log likelihood ratio tests	0.003

*CI, confidence interval; ACC, abdominal aortic calcification. Weighted by: Full sample mobile examination center exam weight. Outcome variable: AAC total 24 score. Exposure variable: serum chloride (mmol/L). Adjusted for age (smooth), gender, race/Hispanic origin, education level, BMI (smooth), systolic blood pressure (smooth), diastolic blood pressure (smooth), total calcium (smooth), cholesterol (smooth), albumin (smooth) and refrigerated serum glucose (smooth)*.

## Discussion

The relationship between serum chloride and aortic calcification has not received enough attention. Some studies have found that the Wnt/β-Catenin pathway is involved in arterial calcification via regulation of the osteoprotegerin)/receptor activator of NF-κ B ligand system. The Wnt/β-Catenin pathway is activated or inhibited by lithium chloride *in vitro* and *in vivo* (22), implying that chloride may be associated with arterial calcification. Chloride is the cause of arterial calcification. Elastin, a structural protein present in abundance in the arterial wall, is prone to calcification in many diseases; meanwhile, aluminum chloride–pretreated elastin has been shown to be completely resistant to calcification (23, 24). However, none of these earlier studies have directly shown that chloride is associated with arterial calcification; moreover, some of the studies were in animal aortas (23, 24). In this study, we therefore aimed to investigate the association between serum chloride and AAC. We found that serum chloride is independently related to the AAC-24. For serum chloride levels lower than 92 mmol/L, the AAC-24 increased as serum chloride increased, indicating increased risk for calcification; however, for serum chloride levels >92 mmol/L, the AAC-24 decreased as serum chloride increased, indicating a protective effect.

Our large sample size allowed subgroup analysis. When refrigerated serum glucose was 5.38–32.03 mmol/L, the smooth fitting curve between serum chloride and AAC-24 was inverted-U shaped, which is similar to the overall fitting curve between serum chloride and AAC-24, but the inflection point was >92 mmol/L. In the low serum glucose group (2.72–5.33 mmol/L), there was a downward fitting curve. In the low age triad (40–50 years), the smooth fitting curve between serum chloride and AAC-24 score was flat. The smooth fitting curves of low systolic blood pressure (74–116 mmHg) and high systolic blood pressure (76–122 mmHg) were flat.

Since we used a nationally representative sample, our findings are generalizable to the whole US population. Our results suggest that serum chloride levels in adults should be maintained at an appropriate level, and that control of serum chloride level could be a new method for the prevention and control of peripheral artery calcification. Appropriate and individualized measures for prevention for AAC may have to be developed for different ethnic populations, sexes, and age-groups.

It is important to recognize the limitations of our research. First, because of the cross-sectional design of our study, a causal relationship between adult serum chloride level and AAC-24 cannot be inferred. Second, confounding factors not analyzed in this study may affect the results. Third, no attempt has been made to clarify the mechanism by which serum chloride affects abdominal aortic calcification. Fourth, it would be more convincing if the range of the lowest and highest values of serum chloride among the participants examined were larger.

## Conclusions

Below a cutoff value of 92 mmol/L, serum chloride appears to be a risk factor for development of abdominal aortic calcification, but serum levels >92 mmol/L may protect against abdominal aortic calcification. Thus, detection of serum chlorine detection could be a simple and accurate method for screening individuals for risk of abdominal aorta calcification and cardiovascular disease.

## Data Availability Statement

The datasets presented in this study can be found in online repositories. The names of the repository/repositories and accession number(s) can be found in the article/Supplementary Material.

## Ethics Statement

This study was approved by the Ethics Review Board of the National Center for Health Statistics. Informed consent was not considered necessary as this was an analysis of a database.

## Author Contributions

SH, JP, and YW contributed to data collection, analysis, and writing of the manuscript. SH, TL, WZ, JY, DZ, YZ, SW, QG, LS, DY, JP, YW, and JX contributed to study design and writing of the manuscript. All authors read and approved the final manuscript.

## Funding

This study was supported by grants from the National Natural Science Foundation of China (81860379 and 82160410) and the Science and Technology Planning Project at the Department of Science and Technology of Jiangxi Province, China (20171BAB 205075).

## Conflict of Interest

The authors declare that the research was conducted in the absence of any commercial or financial relationships that could be construed as a potential conflict of interest.

## Publisher's Note

All claims expressed in this article are solely those of the authors and do not necessarily represent those of their affiliated organizations, or those of the publisher, the editors and the reviewers. Any product that may be evaluated in this article, or claim that may be made by its manufacturer, is not guaranteed or endorsed by the publisher.

## Abbreviations

AAC-24: AAC total 24 score
ACC: abdominal aortic calcification
BMI: body mass index
DXA: dual-energy X-ray absorptiometry
NHANES: National Health and Nutrition Examination Survey.
