# Supplementary material for: Serum Chloride Level Is Associated With Abdominal Aortic Calcification
Source: Front Cardiovasc Med. 2022 Jan 18;8:800458. doi: 10.3389/fcvm.2021.800458 (PMC8805995; doi:10.3389/fcvm.2021.800458)
Supplement: Supplementary file 1 [file Table_1.DOCX]

**Table S1. Detailed calculation process of the AAC-24 scoring method.**

|  | Anterior aortic wall | Posterior aortic wall | Total |
| --- | --- | --- | --- |
| L1 lumbar region | “0” if there was no calcification | “0” if there was no calcification | 0-6 |
|  | “1” if one-third or less of the aortic wall in that segment was calcified | “1” if one-third or less of the aortic wall in that segment was calcified |  |
|  | “2” if more than one-third but less than two-thirds was calcified | “2” if more than one-third but less than two-thirds was calcified |  |
|  | “3” if more than two-thirds was calcified. | “3” if more than two-thirds was calcified. |  |
| L2 lumbar region | “0” if there was no calcification | “0” if there was no calcification | 0-6 |
|  | “1” if one-third or less of the aortic wall in that segment was calcified | “1” if one-third or less of the aortic wall in that segment was calcified |  |
|  | “2” if more than one-third but less than two-thirds was calcified | “2” if more than one-third but less than two-thirds was calcified |  |
|  | “3” if more than two-thirds was calcified. | “3” if more than two-thirds was calcified. |  |
| L3 lumbar region | “0” if there was no calcification | “0” if there was no calcification | 0-6 |
|  | “1” if one-third or less of the aortic wall in that segment was calcified | “1” if one-third or less of the aortic wall in that segment was calcified |  |
|  | “2” if more than one-third but less than two-thirds was calcified | “2” if more than one-third but less than two-thirds was calcified |  |
|  | “3” if more than two-thirds was calcified. | “3” if more than two-thirds was calcified. |  |
| L4 lumbar region | “0” if there was no calcification | “0” if there was no calcification | 0-6 |
|  | “1” if one-third or less of the aortic wall in that segment was calcified | “1” if one-third or less of the aortic wall in that segment was calcified |  |
|  | “2” if more than one-third but less than two-thirds was calcified | “2” if more than one-third but less than two-thirds was calcified |  |
|  | “3” if more than two-thirds was calcified. | “3” if more than two-thirds was calcified. |  |
| Total | 0-12 | 0-12 | 0-24 |
