# Supplementary material for: Serum Chloride Level Is Associated With Abdominal Aortic Calcification
Source: Front Cardiovasc Med. 2022 Jan 18;8:800458. doi: 10.3389/fcvm.2021.800458 (PMC8805995; doi:10.3389/fcvm.2021.800458)
Supplement: Supplementary file 2 [file Table_2.DOCX]

**Table S2. Analysis of threshold effect and saturation effect with 113** **mmol/L as cutoff serum chloride value point.**

| Outcome | AAC Total 24 Score |
| --- | --- |
|  | β (95%CI) P-value |
| Model I |  |
| A straight-line effect | -0.099 (-0.139, -0.059) <0.0001 |
| Model II |  |
| Fold points (K) | 113 |
| < K-segment effect 1 | -0.100 (-0.140, -0.059) <0.0001 |
| >K-segment Effect 2 | 0.321 (-1.108, 1.751) 0.6597 |
| Effect size difference of 2 versus 1 | 0.421 (-1.011, 1.853) 0.5646 |
| Equation predicted values at break points | 0.165 (-0.243, 0.572) |
| Log likelihood ratio tests | 0.580 |

Note: Abbreviations: CI, confidence interval; ACC, abdominal aortic calcification. Weighted by: Full sample mobile examination center exam weight. Outcome variable: AAC total 24 score. Exposure variable: serum chloride (mmol/L). Adjusted for age (smooth), gender, race/Hispanic origin, education level, BMI (smooth), systolic blood pressure (smooth), diastolic blood pressure (smooth), total calcium (smooth), cholesterol (smooth), albumin (smooth) and refrigerated serum glucose (smooth).
